# The Entomopathogenic Fungal Endophytes *Purpureocillium lilacinum* (Formerly *Paecilomyces lilacinus*) and *Beauveria bassiana* Negatively Affect Cotton Aphid Reproduction under Both Greenhouse and Field Conditions

**DOI:** 10.1371/journal.pone.0103891

**Published:** 2014-08-05

**Authors:** Diana Castillo Lopez, Keyan Zhu-Salzman, Maria Julissa Ek-Ramos, Gregory A. Sword

**Affiliations:** 1 Department of Entomology, Texas A&M University, College Station, Texas, United States of America; 2 Department of Immunology and Microbiology, Autonomous University of Nuevo Leon, San Nicolás de los Garza, Nuevo Leon, Mexico; University College Dublin, Ireland

## Abstract

The effects of two entomopathogenic fungal endophytes, *Beauveria bassiana* and *Purpureocillium lilacinum* (formerly *Paecilomyces lilacinus*), were assessed on the reproduction of cotton aphid, *Aphis gossypii* Glover (Homoptera:Aphididae), through *in planta* feeding trials. In replicate greenhouse and field trials, cotton plants (*Gossypium hirsutum*) were inoculated as seed treatments with two concentrations of *B. bassiana* or *P. lilacinum* conidia. Positive colonization of cotton by the endophytes was confirmed through potato dextrose agar (PDA) media plating and PCR analysis. Inoculation and colonization of cotton by either *B. bassiana* or *P. lilacinum* negatively affected aphid reproduction over periods of seven and 14 days in a series of greenhouse trials. Field trials were conducted in the summers of 2012 and 2013 in which cotton plants inoculated as seed treatments with *B. bassiana* and *P. lilacinum* were exposed to cotton aphids for 14 days. There was a significant overall effect of endophyte treatment on the number of cotton aphids per plant. Plants inoculated with *B. bassiana* had significantly lower numbers of aphids across both years. The number of aphids on plants inoculated with *P. lilacinum* exhibited a similar, but non-significant, reduction in numbers relative to control plants. We also tested the pathogenicity of both *P. lilacinum* and *B. bassiana* strains used in the experiments against cotton aphids in a survival experiment where 60% and 57% of treated aphids, respectively, died from infection over seven days versus 10% mortality among control insects. Our results demonstrate (i) the successful establishment of *P. lilacinum* and *B. bassiana* as endophytes in cotton via seed inoculation, (ii) subsequent negative effects of the presence of both target endophytes on cotton aphid reproduction using whole plant assays, and (iii) that the *P. lilacinum* strain used is both endophytic and pathogenic to cotton aphids. Our results illustrate the potential of using these endophytes for the biological control of aphids and other herbivores under greenhouse and field conditions.

## Introduction

Fungal endophytes can protect plants from a wide range of stressors including insect pests [1]. In this study we refer to an endophyte as defined by Schulz (2005) [2] to be microorganisms (fungi or bacteria) found in asymptomatic plant tissues for all or part of their life cycle without causing detectable damage to the host. The need for the development of new strategies for the control of agricultural insect pests continues to increase due to factors such as development of insecticide resistance [3]–[5]. Here we focus on entomopathogenic fungal endophytes [6] and the ecological role these fungi can play in agricultural systems.

Entomopathogenic fungal endophytes have been isolated from a variety of different plant species and tissues, and can be inoculated to establish endophytically in a range of other plants to test for adverse effects, if any, on different insect herbivores [1] [6]–[7]. These entomopathogenic fungal endophytes are classified as non-clavicipitaceous [8]; referring to fungal endophytes that are usually horizontally transmitted. Clavicipitaceous endophytes, on the other hand, are found in grasses and are typically vertically transmitted, potentially leading to an obligate relationship and higher infection rates with their hosts [8]–[9]. Clavicipitaceous endophytes, named true endophytes, have been studied more extensively than non-clavicipitaceous species and are generally considered mutualistic. Evidence suggests that these fungal endophytes can significantly improve host plant tolerance to drought, insects, diseases, and nematodes, and in exchange, plants provide protection, nutrition and dissemination of the fungi [10].

A number of benefits to plants are also conferred by non-clavicipitaceous endophytes [9] [11]–[14]. As endophytes, several non-clavicipitaceous entomopathogens including *Beauveria bassiana*, *Lecanicillium lecanii, Metharizium anisoplae* and *Isaria spp*. can have negative effects on insect pests when *in planta*, antagonize plant pathogens and promote plant growth [6] [15]. The activity of *B. bassiana* has received particular attention due to its negative effects on a variety of insect herbivores including the cotton aphid [7] [16]–[22].

The fungus *P. lilacinum* is more widely known as *Paecilomyces lilacinus*, having undergone a recent taxonomic revision [23]. To our knowledge there are no studies demonstrating *P. lilacinum* as an endophytic fungus causing negative effects on insect herbivores, but there are reports of it being pathogenic to a number of insects including *Ceratitis capitata*, *Setora nitens*, *A. gossypii*, and *Triatoma infestans* [24]–[28]. Both *B. bassiana* and *P. lilacinum* are commercially available for use as biocontrol agents, but *P. lilacinum* is mainly considered to be a nematophagous, egg-parasitizing fungus, specifically against root-knot nematode, *Meloidogyne incognita*, and several other nematode species including *Radopholus similis*, *Heterodera spp*, *Globodeera spp* [29]–[32].

Cotton aphids, *A. gossypii*, have a broad range of host plants including cultivated cotton, causing damage directly by plant feeding and indirectly through virus transmission and physical contamination of cotton by honeydew production [33]. Most commonly, *A. gossypii* is considered a mid- to late-season pest in cotton. However, extensive use of insecticides such as pyrethroids can decrease its natural enemy community, thereby contributing to the establishment of the aphid as a season-long pest across cotton production areas [34]–[35]. Chronic insecticide use for aphid control has also increased its resistance to several classes of insecticides [36]–[38]. Considering the increasing need for alternative insect management strategies in agricultural systems, we investigated the effects of two entomopathogens, *B. bassiana* and *P. lilacinum*, on the cotton aphid when present endophytically in cotton. Specifically, we tested: 1) the ability of *B. bassiana* and *P. lilacinum* to establish as endophytes in cotton seedlings when inoculated at the seed stage, and 2) the effects of these endophytes on cotton aphid reproduction using *in planta* feeding trials in both greenhouse and field environments.

## Materials and Methods

### Plants and endophytic fungi strains

The cotton seeds used for all experiments were variety LA122 (All-Tex Seed, Inc.). The *P. lilacinum* strain was isolated from a field survey of naturally-occurring fungal endophytes in cotton [39]. This strain was confirmed to be *P. lilacinum* (formerly *P. lilacinus*) by diagnostic PCR and subsequent sequencing of the ribosomal ITS region using specific species primers [40]. The *B. bassiana* was cultured from a commercially obtained strain (Botanigard, BioWorks Inc, Victor, NY). Stock spore solutions of each fungus were made by adding 10 ml of sterile water to the fungi cultured on potato dextrose agar (PDA) in 10 cm diameter petri dish plates and scraping them with a sterile scalpel. The resulting mycelia and spores were then filtered through cheese cloth into a sterile beaker. A haemocytometer was used to calculate the conidia concentrations of the resulting stock solutions. Final treatment concentrations were reached by dilution using sterile water.

### Cotton seed inoculation

Seeds were surfaced sterilized prior to soaking in different spore concentrations by immersion in 70% ethanol for 3 minutes with constant shaking, then 3 minutes in 2% sodium hypochlorite (NaOCl) followed by three washes in sterile water, based on Posada et al. [18]. The third wash was plated on PDA media to confirm surface sterilization efficiency. Seeds were then soaked for 24 hours in two different spore concentrations of the two fungi and sterile water was used as control. Spore concentrations for each fungus were zero (control), 1×10^6^ spores/ml (treatment 1) and 1×10^7^ spores/ml (treatment 2) based on inoculum concentrations used in previous studies of endophytic entomopathogens [7] [17]–[18] [22] [70]. Beakers containing the seeds were placed in a dark environment chamber at 28°C until the next day for planting. Soaked seeds were planted in individual pots (15 cm diameter) containing unsterilized Metro mix 900 soil consisting of 40–50% composted pine bark, peat moss, vermiculite, perlite and dolomitic limestone (Borlaug Institute, Texas A&M). All plants were grown in a greenhouse at ∼25°C with natural photoperiod for the duration of the experiment. Pots were placed in a complete randomized design, watered as needed, and no fertilizer was applied throughout the experiments.

### Confirmation of plant colonization by endophytic fungi

We have no reason to assume that 100% of the endophyte-treated plants are always colonized by the endophytes when inoculated as seed treatments. Given this constraint, we decided to use two detection methods simultaneously, PDA culturing and diagnostic PCR analysis, to positively confirm the presence of the target endophytes in the experimental plants from the greenhouse experiments, but not for our field experiments. At the end of each greenhouse trial, all treated and control plants were harvested, and each plant was cut in half longitudinally using a sterile scalpel. Fragments of leaves of 1 cm^2^, stems and roots of 1 cm length were plated on PDA media and placed in growth chamber at 28°C to check for presence of the endophytes. The other half of the plant was freeze dried and DNA was extracted utilizing the CTAB protocol [41]. Species specific oligonucleotide primers for *B. bassiana* 5′CGGCGGACTCGCCCCAGCCCG 3′, 3′ CCGCGTCGGGGTTCCGGTGCG 5′ [39] and *P. lilacinum* 5′ CTCAGTTGCCTCGGCGGGAA 3′, 3′ GTGCAACTCAGAGAAGAAATTCCG 5′ [40] (Sigma-Aldrich, Inc St Louis, MO) were used for diagnostic PCR assays. PCR products were visualized on a 2% agarose gel to determine the presence of the inoculated fungal endophytes based on amplification of a DNA fragment of the expected size (positive control). Given the larger size of the plants utilized in our field trials and the impracticality of PDA plating and extracting genomic DNA from entire large plants, we did not test for the presence of the target endophytes in the experimental plants. Instead, we analyzed our data as treatment groups [control, *B. bassiana* (10^6^), *B. bassiana* (10^7^), *P. lilacinum* (10^6^) and *P. lilcainum* (10^7^)] with concentration effects nested within endophyte treatment and present our results as such.

### Cotton aphid reproduction tests

A colony of *A. gossypii* was maintained on caged cotton plants in the same greenhouse as the experimental plants as described above. For all endophyte-aphid greenhouse trials, second instar nymphs were placed directly on to the experimental control and endophyte-treated cotton plants. Experimental and control plants with aphids were placed in individual clear plastic cages of 45 cm height and 20 cm diameter, then sealed on top with no-see-um mesh (Eastex products, NJ) to avoid aphid escape or movement between plants.

### *B. bassiana* cotton aphid greenhouse experiments

Greenhouse assays of the effects of endophytic *B. bassiana* on cotton aphid reproduction consisted of three independent tests, each utilizing slightly different protocols. The first was initiated when plants were 13 days old (1^st^ true leaf stage) with aphids allowed to feed for seven days on 10 plants per treatment group. For the second trial, we used older plants (20 days old/third true leaf stage) and aphids were left to reproduce for a longer period of time (14 days) on 10 plants per treatment. At the end of each trial, total aphid numbers were recorded on each individual plant. The third independent test consisted of only a single reproduction trial in which ten 2^nd^ instar aphids were placed on 15 day old plants (second true leaf stage) and left to reproduce 14 days on 15 plants per treatment group, but the cohorts of aphids on each plant were sampled twice at 7 and then again at 14 days.

### *P. lilacinum* cotton aphid greenhouse experiments

We conducted two replicate experiments testing for effects of endophytic *P. lilacinum* on cotton aphid reproduction utilizing the same reproduction test protocol for each trial. In these trials, ten 2^nd^ instar aphids were left to reproduce on the same plants for 14 days consecutively and sampled twice at 7 and then again at 14 days. Ten 1^st^ true leaf stage plants per treatment group were utilized for the first trial; 15 plants per treatment group were used for the second trial.

### Cotton aphid field trials for both *B. bassiana* and *P. lilacinum*

During the summers of 2012 and 2013, experimental field trials were conducted at the Texas A&M University Field Station located near College Station in Burleson, Co., TX (N 30° 26′ 48′′ W 96° 24′ 05.12′′) at an elevation of 68.8 m. We utilized a randomized block design with five seed inoculation treatments (T1: Control, T2: *B. bassiana* 1×10^6^, T3: *B. bassiana* 1×10^7^, T4: *P. lilacinum* 1×10^6^ and T5: *P. lilacinum* 1×10^7^). Surface sterilized seeds were inoculated with the different treatments as described in our greenhouse assay protocol. Treatments were replicated six times, making a total of 30 plots in the field. Each plot was comprised of 4 rows of 16.6 m length and planted with 15 seeds per meter. For the aphid reproduction experiments, we utilized the same protocol during both field seasons whereby a total of 75 cone shaped metal framed cages (0.35 m of height) were randomly assigned to be placed over endophyte-inoculated and control plants (15 cages/treatment) and set up on May 17, 2012 and June 24, 2013, respectively (delayed experiment due to rain in 2013). Predators were eliminated if found prior to enclosing the caged plants with no-see-um mesh (Eastex products, NJ) to prevent aphid escapes and entrance of predators. Ten second instar aphid nymphs from the laboratory colony were placed on each plant and left to reproduce for 14 days. At the end of the experiment, cages were removed, the entire plant was bagged and brought back to laboratory for total aphid number counts.

### Fungal pathogenicity experiment

To assess pathogenicity of both the *P. lilacinum* strain recovered in our endophyte survey of cotton [39], and the commercial *B. bassiana* strain utilized in our endophyte trials, we performed a cotton aphid survival experiment as per Gurunlingappa et al. [22] and Vega et al. 2008 [70] with slight modification. The same spore concentrations used in our endophyte *in planta* experiment were used for this test for both endophytes (0, 1×10^6^ and 1×10^7^ spores/ml). Thirty 2^nd^ instar aphids per treatment were dipped in spore solutions for 5 seconds, and then placed on fresh cotton leaves kept on moistened filter paper (to prevent drying out) inside 10 cm diameter petri dishes sealed with parafilm (Bemis flexible packaging, Neenah, WI). Ten aphids per petri dish were placed in three replicate petri dishes per treatment. Aphids were checked daily for mortality and dead aphids were removed, plated and incubated on PDA media to confirm emergence of the entomopathogens from aphid cadavers.

### Statistical analyses

All data were tested for normality assumptions using a qqplot, Levene's homogeneity test and the Shapiro-Wilk normality test at alpha = 0.05 significance level. For the first independent *B. bassiana* greenhouse experiment, ANOVA and t-tests were performed to compare aphid reproduction differences among plants after 7 days of feeding. In the second and third B. *bassiana* tests, the data were non-normal and nonparametric Kruskal-Wallis and Mann-Whitney U tests were used. For both *P. lilacinum* greenhouse trials, a repeated measures ANOVA was performed with time as a repeated factor to test for differences in aphid numbers between plants after 7 and 14 days of reproduction because aphids on the same plants were sampled sequentially. Aphid field trials for both 2012 and 2013 were analyzed using ANOVA followed by pairwise comparisons (control vs. treatment). We conducted a combined ANOVA analysis of the field data across both 2012 and 2013 to test for year, treatment, and year by treatment effects. For the cotton aphid pathogenicity experiment, a Kaplan-Meier survival analysis was performed to compare the cumulative survival of treated vs. untreated control aphids. All analyses were conducted using SPSS 22 (IBM SPSS, Armonk NY).

## Results

### Plant colonization by endophytic fungi

Our culturing results showed no fungal growth on the PDA plating of the third sterile water wash of either the surface sterilized seeds or plant samples, indicating the efficacy of our surface sterilization. Thus, we assume that the fungi growing in the media from surface-sterilized plant materials were endophytes that came from within plant tissues and not epiphytes from the plant surface. Utilizing combined PDA plating and diagnostic PCR detection methods revealed 30–45% more instances of positive endophytic colonization relative to PDA plating alone. *B. bassiana* was detected in 35% and 55% of the treated plants in the first (7 day) and second (14 day) greenhouse trials, respectively. For the third *B. bassiana* trial which consisted of using the same plants for both measurements of aphid reproduction at 7 and 14 days, B. *bassiana* was detected in 53.3% of the treated plants. In the *P. lilacinum* experiments, the target endophyte was detected in 55% and 45% of plants in the first and second trials, respectively.

### *B. bassiana* cotton aphid greenhouse experiments

**Figure 1:**
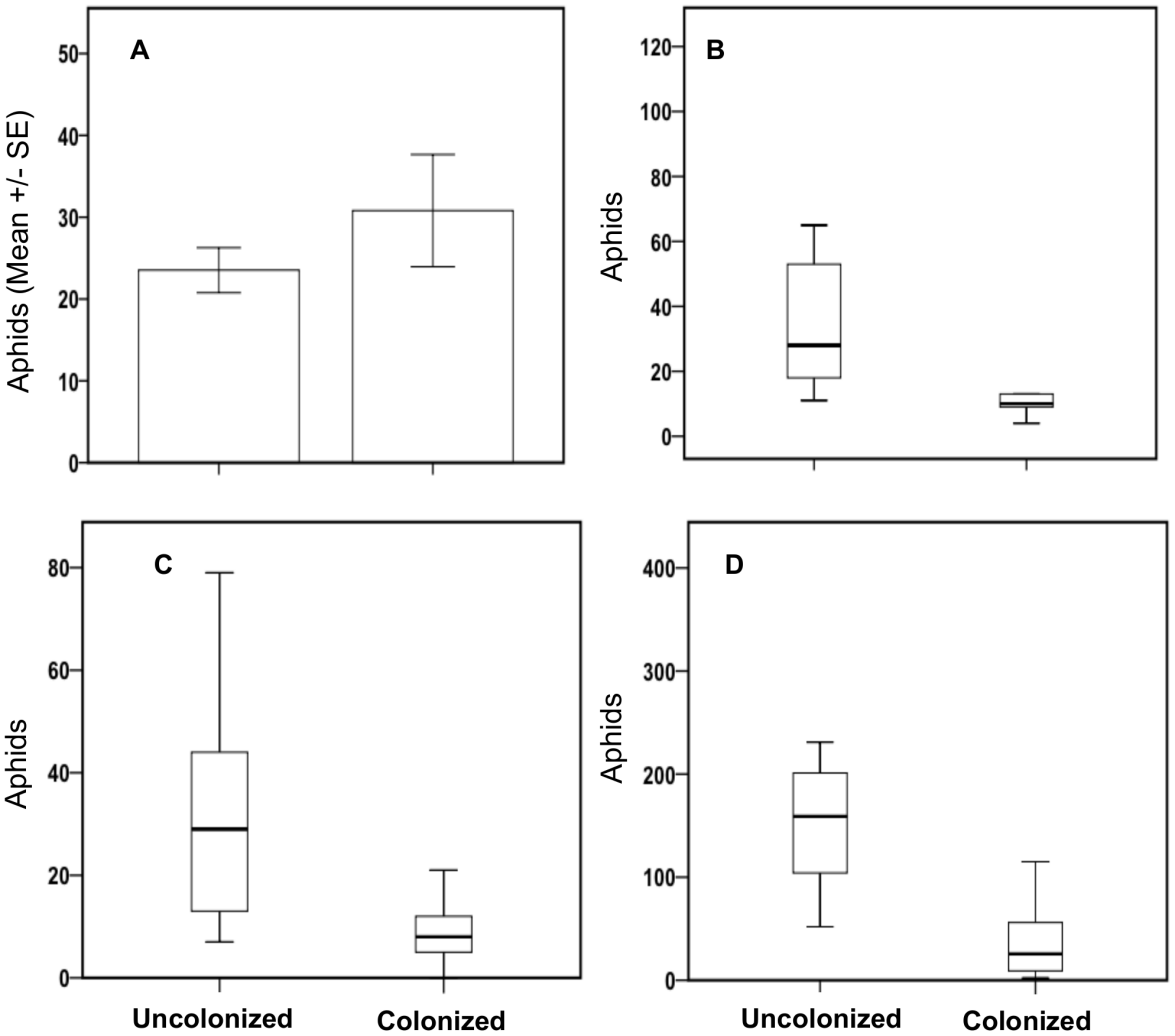
Effects of endophytic *B. bassiana* on cotton aphid reproduction in three independent greenhouse assays. Cotton aphid reproduction on plants positively colonized by endophytic *B. bassiana* versus uncolonized plants after (a) 7 days in the first trial, (b) 14 days in the second trial, and (c) 7 and (d) 14 days successively in the third trial.

Our results were analyzed both as treatments (control, low and high concentration) and by confirmed positive colonization of plants by the target endophyte (colonized vs. uncolonized). In the first test, the mean number of cotton aphids per plant on *B. bassiana* treated plants was not significantly different from those on control plants after 7 days of reproduction when analyzed by treatment groups (F = 2.07; df = 2,29; P = 0.145), but was significantly different when analyzed by positive colonization of the endophyte (t-test; P = 0.014) (Fig 1a). In the second test, we observed a significant negative effect on reproduction of cotton aphids after 14 days when analyzed by treatment groups (Kruskal-Wallis = 6.744; P = 0.034) as well as by positive colonization of the endophyte (Mann Whitney U = 44; P = 0.004) (Fig 1b). In our third *B. bassiana* trial, there was no significant effect on the number of aphids per plant after 7 days when analyzed by treatment (Kruskal-Wallis = 4.74; P = 0.093), but there was a significant effect on aphids when analyzed by positive colonization by the endophyte (Mann-Whitney U = 60.50; P = <0.0001) (Fig 1c). Similarly at the end of the 14 days in the same experiment, there were no significant effects on the number of aphids when the data were analyzed by treatment (Kruskal Wallis = 3.069; P = 0.216), but a significant effect was observed when the data were analyzed by plant positive colonization by the endophyte (Mann Whitney U = 58; P<0.0001) (Fig 1d).

### *P. lilacinum* cotton aphid greenhouse experiments

**Figure 2:**
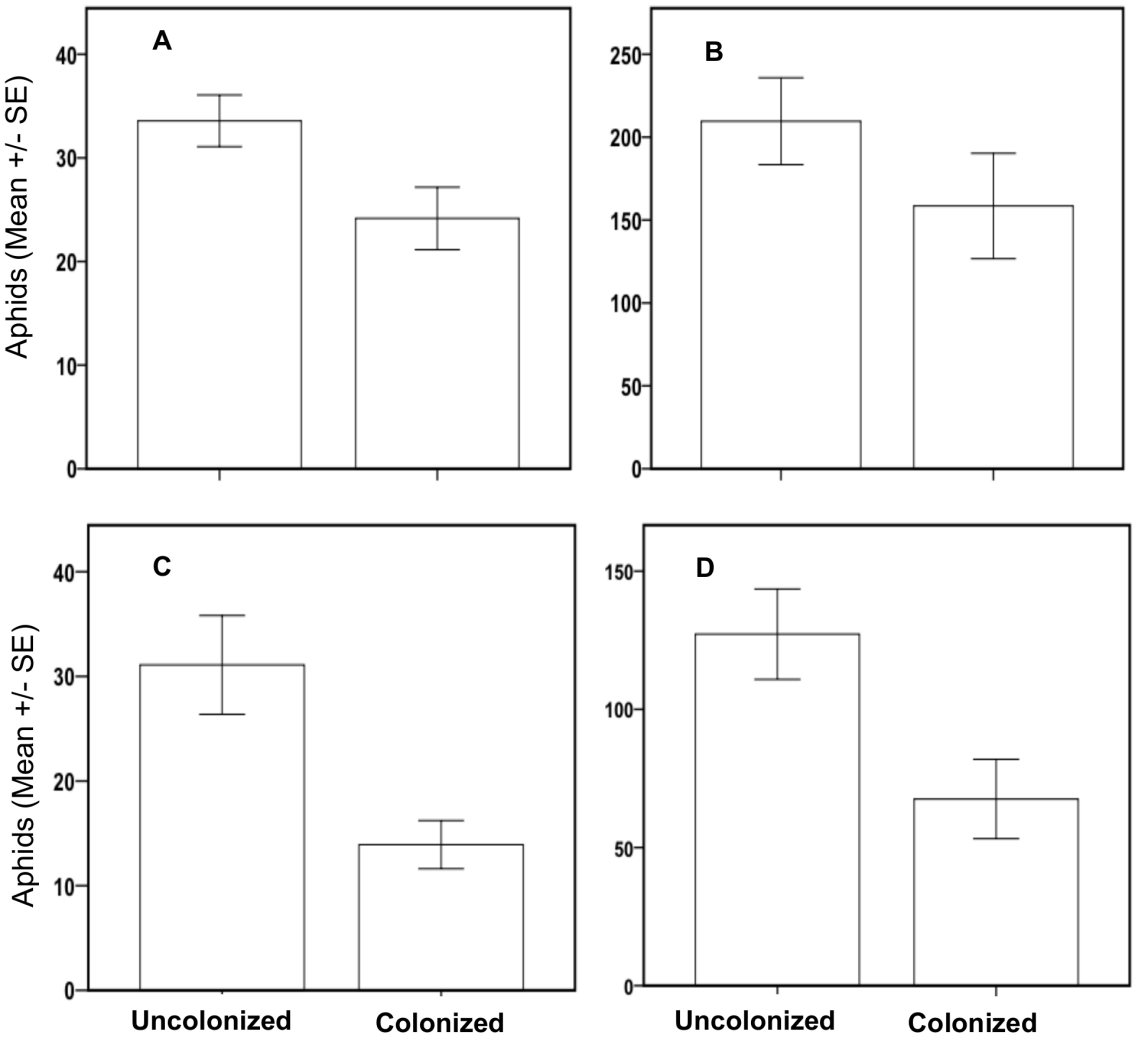
Effects of endophytic *P. lilacinum* on cotton aphid reproduction in two replicate greenhouse assays. Cotton aphid reproduction on plants positively colonized by endophytic *P. lilacinum* versus control plants after 7 days in the first (a) and second (c) trials, followed by 14 days in the same trials (b & d, respectively).

As with the *B. bassiana* trials above, we present the results of analyses categorizing the data as both treatment groups and positive versus negative colonization. In the first *P. lilacinum* trial, aphid numbers varied significantly with time (Repeated Measures ANOVA F = 60.40; df = 1,28; P = 0.0001), but no significant endophyte treatment effect was observed when data were analyzed by plant positive colonization (F = 0.026; df = 1,28; P = 0.873). However, when analyzed based on treatment groups, there was a significant effect of time (F = 69.56; df = 1,27; P<0.0001) as well as endophyte treatment (F = 140.48; df = 2,27; P = 0.049) (Fig 2a). After increasing our sample size in the second trial, we observed a significant effect of both time (F = 53.73; df = 1,42;P = 0.0001) and treatment when analyzed based on plant positive colonization by the endophyte (F = 8.05; df = 1,42; P = 0.007) (Fig 2c). Although there was a significant effect of time (F = 52.52; df = 1,41; P<0.000) on the number of aphids when we analyzed our data by treatment groups (control, low or high concentration), the effect of endophyte treatment was not significant (F = 0.546; df = 241; P = 0.583).

### Cotton aphid field trials of both *B. bassiana* and *P. lilacinum*

**Figure 3:**
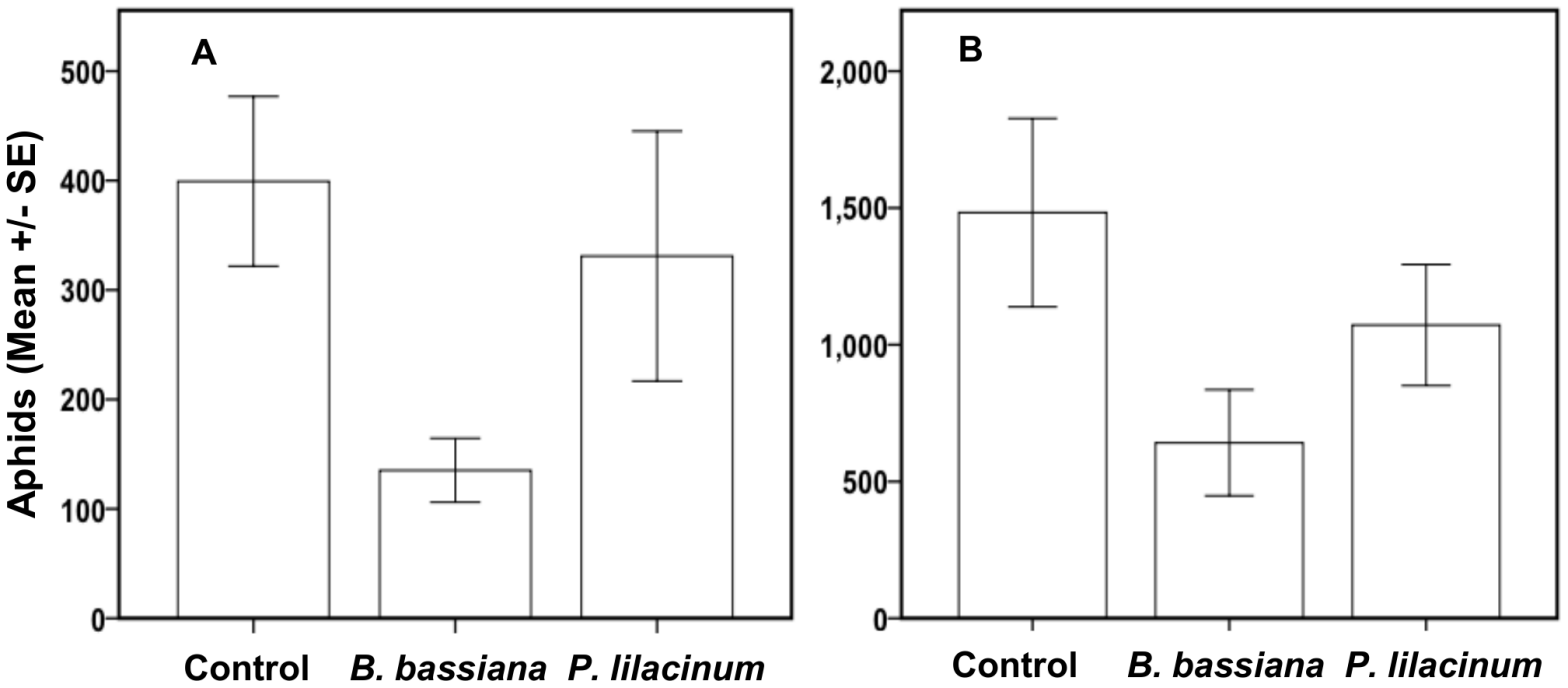
Effects of endophytic *B. bassiana* and *P. lilacinum* on cotton aphid reproduction under field conditions. Cotton aphid reproduction after 14 days on plants inoculated as seeds with either *B. bassiana* or *P. lilacinum* versus uninoculated control plants under field conditions in (a) 2012 and (b) 2013.

In both 2012 and 2013 there was no effect of seed treatment spore concentration within each endophyte treatment (2012 Nested ANOVA, F = 1.95; df = 2,77; P = 0.149 and 2013 Nested ANOVA F = .935; df = 2,67; P = 0.398), therefore data from both concentrations were grouped for each endophyte in subsequent analyses. Across both years of the field trial, there was a significant effect of endophyte treatment (ANOVA, F = 7.31; df = 5,132; P = 0.001) and also a significant year effect (ANOVA, F = 17.43; df = 5,132; P<0.0001), but no endophyte by year interaction (ANOVA, F = 0.547; df = 5,132; P = 0.580). During the summer of 2012, there was a significant overall effect of endophyte treatment on the number of cotton aphids per plant at the end of 14 days of reproduction (ANOVA, F = 4.12; df = 2,73; P = 0.02). Follow-up pairwise comparisons revealed that there were significantly fewer aphids on cotton plants from *B. bassiana*-treated vs. control plots (P  = 0.006). The difference in aphid numbers on plants in *P. lilacinum*-treated vs. control plots exhibited a similar but non-significant reduction (P = 0.085) (Fig 3a). Similarly in 2013, there was a significant overall effect of endophyte treatment on aphid reproduction at the end of 14 days (ANOVA, F = 3.13; df = 2,59; P = 0.05). Pairwise comparisons indicated that inoculation of plants with *B. bassiana* had a significant negative effect on aphid reproduction vs. control (P = 0.016), but only a non-significant trend was observed with *P. lilacinum* vs. the control (P = 0.086) (Fig 3b).

### Cotton aphid survival experiment

There was no significant difference in aphid mortality between those treated with two different concentrations (1×10^6^ or 1×10^7^) of conidia solutions of each fungus. Thus, the data from both concentrations were pooled and analyzed together for each fungus. There was a highly significant increase in mortality between aphids treated with either *P. lilacinum* (60%) or *B. bassiana* (57%) vs. the controls (10%) (Kaplan-Meier, P<0.0001 for both fungi).

## Discussion

Our results provide the first report of the negative effects of two endophytic entomopathogenic fungi, *B. bassiana* and *P. lilacinum*, on cotton aphid reproduction when feeding on whole intact cotton plants inoculated as seed treatments. Importantly, we observed negative effects under both greenhouse and field conditions. We also provide the first evidence for an endophytic effect of *P. lilacinum* on herbivorous insect performance.

After analyzing our data based on positive plant colonization by the target endophyte, we found that aphid reproduction on cotton plants positively colonized by *B. bassiana* was reduced in three independent greenhouse trials. Although the results of our first trial testing the effects of *P. lilacinum* as an endophyte on aphid reproduction revealed only a significant effect of time but not treatment, we attributed this to a small sample size for the given effect size based on the results of power analysis (Power = 0.175) (Fig. 2b). After increasing the sample size in the second *P. lilacinum* trial, we observed a significant effect of both time and treatment on the reproduction of cotton aphid with lower aphid numbers on endophyte-colonized plants (Figs. 2c & 2d). Our greenhouse endophyte trial results using *A. gossypii* are similar to those of Martinuz et al. [42] in which whole squash plants were inoculated with *Fusarium oxysporum* as an endophyte via soil drench, resulting in negative effects on *A. gossypii* choice and performance. Similarly, Akello et al. [43] showed that *Aphis fabae* feeding on bean plants colonized independently by strains of either *B. bassiana*, *Trichoderma asperellum* or *Gibberella moniliformis* reproduced poorly compared to those on control plants. Both Martinuz et al. [42] and Akello et al [43] attribute the negative effects on aphid fitness to be due to chemical changes in the plant that were systemically induced by the presence of the endophyte, though the specific mechanism by which these fungi activated a systemic response within the plants was not investigated.

The ability of *B. bassiana* to establish as an endophyte across a range of plants has been well established [e.g., cotton, corn, bean, wheat, pumpkin, tomato [7]; coffee [18]); sorghum [44]; banana [19]; tomato [20]; jute [21] and pine [45]. A number of plant-endophyte-insect interaction experiments, including a cotton aphid study by Gurunlingappa et al. [7] have been performed using cut leaf bioassays rather than whole intact plant experiments [25] [46]–[48]. Utilizing leaf cuts rather than whole intact plants can potentially cause release of allelochemicals due to direct plant damage that may have negative effects on insects that could obscure those caused by the presence of an endophyte [49]. Alternatively, cutting plants and abscising leaves may induce changes in plant chemistry that alter the interaction between the endophyte and host in ways not observed in intact plants [49]. Demonstrations of negative effects of endophytic entomopathogens including *B. bassiana* on herbivores in more natural whole plant feeding assays are relatively rare, but have been shown for a few species including aphids [42]–[43]. Similarly, there are only a few examples of negative effects on lepidopteran species caused by endophytic colonization by *B. bassiana* using whole plant assays including *Ostrinia nubilalis* and *Helicoverpa zea* [16] [20].

To our knowledge, there are no reports in the literature of negative endophytic effects of *P. lilacinum* on herbivorous insects. This is not surprising since this fungus was until recently thought to mainly have pathogenic properties against nematodes and not insects. Historically, *P. lilacinum* has been considered largely as a soil-born nematode egg parasite and used as a biocontrol agent against nematode pests such as root-knot, *Meloidogyne incognita*, and reniform, *Rotylenchulus reniformis*, nematodes [50]–[52]. However, recent evidence indicates that *P. lilacinum* can also be an entomopathogen [24]–[28]. Our results indicate that the *P. lilacinum* strain isolated from cotton by Ek-Ramos et al. [39] can negatively affect insect herbivores when present as an endophyte and that it is also pathogenic to insects. Interestingly, the same strain has also been observed to parasitize root-knot nematode eggs in simple lab bioassays and negatively affect nematode reproduction when present as an endophyte in *in planta* assays (W. Zhou, J.T. Starr and G.A. Sword, unpublished results).

The mechanisms by which herbivores can be negatively affected by clavicipitaceous obligate endophytes have been studied in a few different grass species and can vary from antixenosis and/or antibiosis mediated by constitutive production and or induction of secondary compounds produced by the plant [53]–[55] or secondary metabolites produced by the endophytes themselves [13] [22] [56]–[61] [64]. It is important to mention that infection rates of natural populations of grasses by these endophytes can vary depending on the genetic and environmental background of the population and these factors can determine if this symbiosis goes from mutualistic to antagonistic [63]–[67]. Another hypothesis for the mechanism by which endophytes can negatively affect herbivores is based on the idea that endophytes can alter the phytosterol profiles of plants and compete with insects for these compounds which are essential for their development [46] [62]. The mechanisms by which entomopathogenic endophytic fungi may protect plants from insect herbivores are unknown. Although these endophytes do produce secondary metabolites [22] [68], we do not know if this is the main cause for the negative effects on aphids when feeding on endophytically-colonized plants observed in our study. The literature also suggests a systemic response in the plant can be induced by the presence of some entomopathogenic endophytes including *B. bassiana* that confers resistance against plant pathogens [68]–[69]. Whether an induced systemic response accounts for the negative effects on insects observed in our study remains to be determined.

The mode of establishment and duration of presence of endophytic fungi in plants varies among the different plant-endophyte combinations tested to date [7] [17]–[21] [44]–[45]. In some cases, intentionally inoculated endophytes can be retained within plants for considerable amounts of time, including *B. bassiana* found for as long as eight months in coffee [18] or nine months in *Pinus radiata* [45]. Our study indicates that *B. bassiana* and *P*. *lilacinum* were still present in cotton plants up to 34 days following inoculation as a seed treatment. This duration does not necessarily indicate that *B. bassiana* and *P*. *lilacinum* can only be present in cotton as endophytes for this period of time, but rather that we did not test for the presence/absence of the endophytes beyond 34 days. The average recovery success of the target endophytes used in our studies ranged from 35–55%. Though not a high colonization frequency, we were still able to detect negative effects on aphids feeding on plants colonized by the endophytes. We have not yet rigorously studied the endophytic colonization of cotton by *P. lilacinum* and *B. bassiana*, but *P. lilacinum* was primarily detected in the root tissues whereas *B. bassiana* was found mostly in the above ground tissues. Fungal endophytes are known to occur throughout an entire plant including leaves, stems, roots and reproductive parts, however, tissue specific presence in plants is not required for negative effects on target herbivores. For example, endophytic fungi inhabiting roots can negatively affect the performance and fitness of caterpillars feeding on above ground tissues [13], [71]. Our results support this scenario given that *P. lilacinum* negatively affects aphids feeding on cotton leaves above ground, but is recovered more commonly from below ground root tissues.

The manipulation of endophytic fungi, many of which are completely unstudied, has the potential to protect plants from insect herbivores and other stress factors [1]. We have provided novel evidence showing that the endophytic establishment in cotton of the entomopathogens *B. bassiana* and *P. lilacinum* when inoculated as seeds can adversely affect cotton aphid reproduction not only in greenhouse assays, but also under field conditions. Although we observed a significant year effect, this was due to differences in the total aphid numbers across years (Fig. 3a&b). Importantly, there was no year by endophyte treatment interaction effect. Our field results exhibited the same pattern of negative effects of endophytes on cotton aphids across years in both 2012 and 2013. The consistency of results across years under field conditions that can vary in variety of uncontrolled environmental variables (e.g. precipitation and temperature regimes) is particularly encouraging for the potential reliability of incorporating fungal endophyte manipulations into IPM strategies. Future directions of our work include testing these entomopathogenic endophytes against other insect and nematode herbivores along with phytohormone and transcriptomic analysis to investigate the mechanisms by which these endophytes confer protection to their plant hosts.
